# Author Correction: Evaluation of reopening strategies for educational institutions during COVID-19 through agent based simulation

**DOI:** 10.1038/s41598-021-00201-0

**Published:** 2021-10-13

**Authors:** Ujjal K. Mukherjee, Subhonmesh Bose, Anton Ivanov, Sebastian Souyris, Sridhar Seshadri, Padmavati Sridhar, Ronald Watkins, Yuqian Xu

**Affiliations:** 1 Department of Business Administration, Gies College of Business, University of Illinois at Urbana Champaign, Urbana, IL 61801 USA; 2 Department of Electrical and Computer Engineering, Grainger College of Engineering, University of Illinois at Urbana Champaign, Urbana, IL 61801 USA; 3 School of Information, University of California Berkeley, Berkeley, CA 94720 USA

Correction to: *Scientific Reports* 10.1038/s41598-021-84192-y, published online 17 March 2021

The original version of this Article contained errors in the text, where the RT-qPCR tests were incorrectly referred to as LAMP tests.

In the Introduction,

“In the case of the UIUC SHIELD program, results from the LAMP tests are being made available within 6 to 12 h for upwards of 10K daily tests.”

now reads:

“In the case of the UIUC SHIELD program, results from the RT-qPCR tests are being made available within 6 to 12 h for upwards of 10K daily tests.”

“The rapid saliva-based LAMP tests with an average turnaround time of 6–12 h (e.g., the testing mechanism of the UIUC SHIELD program) are ideally suited for this task.”

now reads:

“The rapid saliva-based tests with an average turnaround time of 6–12 h (e.g., the testing mechanism of the UIUC SHIELD program) are ideally suited for this task.”

In the Results and discussions section, under the subheading ‘Bulk testing capacity’,

“To make our simulations more realistic we assumed that the tests have a sensitivity of 0.92. See Wyllie et al.^22,36^ that reports the sensitivity of saliva-based LAMP tests to be between 0.90 and 0.95.”

now reads:

“To make our simulations more realistic we assumed that the tests have a sensitivity of 0.92. See Wyllie et al.^22,36^ that reports the sensitivity of saliva-based tests to be between 0.90 and 0.95.”

Under the subheading ‘Test sensitivities’,

“Our final study seeks to understand the impact of the sensitivity of tests on the infection mitigation strategy. Early reports^22,36^ claim saliva-based LAMP tests to have an average sensitivity of 92%, i.e., they are able to correctly detect 92% of the cases that are COVID-positive.”

now reads:

“Our final study seeks to understand the impact of the sensitivity of tests on the infection mitigation strategy. Early reports^22,36^ claim saliva-based RT-qPCR tests to have an average sensitivity of 92%, i.e., they are able to correctly detect 92% of the cases that are COVID-positive.”

The original Article has been corrected.

